# Radiotracers for Molecular Imaging of Angiotensin-Converting Enzyme 2

**DOI:** 10.3390/ijms25179419

**Published:** 2024-08-30

**Authors:** Wenqi Xu, Sigrid A. Langhans, David K. Johnson, Erik Stauff, Vinay V. R. Kandula, Heidi H. Kecskemethy, Lauren W. Averill, Xuyi Yue

**Affiliations:** 1 Department of Radiology, Nemours Children’s Health, Delaware, Wilmington, DE 19803, USA; wenqi.xu@nemours.org (W.X.); erik.stauff@nemours.org (E.S.); vinay.kandula@nemours.org (V.V.R.K.); heidi.kecskemethy@nemours.org (H.H.K.); lauren.averill@nemours.org (L.W.A.); 2 Diagnostic & Research PET/MR Center, Nemours Children’s Health, Delaware, Wilmington, DE 19803, USA; sigrid.langhans@nemours.org; 3 Division of Neurology, Nemours Children’s Health, Delaware, Wilmington, DE 19803, USA; 4 Computational Chemical Biology Core, Molecular Graphics and Modeling Laboratory, University of Kansas, Lawrence, KS 66047, USA; dkjohnson@ku.edu

**Keywords:** renin angiotensin system, angiotensin-converting enzymes 2, SARS-CoV-2, COVID-19, molecular imaging, radiotracer, positron emission tomography, single photon emission computed tomography

## Abstract

Angiotensin-converting enzymes (ACE) are well-known for their roles in both blood pressure regulation via the renin-angiotensin system as well as functions in fertility, immunity, hematopoiesis, and many others. The two main isoforms of ACE include ACE and ACE-2 (ACE2). Both isoforms have similar structures and mediate numerous effects on the cardiovascular system. Most remarkably, ACE2 serves as an entry receptor for SARS-CoV-2. Understanding the interaction between the virus and ACE2 is vital to combating the disease and preventing a similar pandemic in the future. Noninvasive imaging techniques such as positron emission tomography and single photon emission computed tomography could noninvasively and quantitatively assess in vivo ACE2 expression levels. ACE2-targeted imaging can be used as a valuable tool to better understand the mechanism of the infection process and the potential roles of ACE2 in homeostasis and related diseases. Together, this information can aid in the identification of potential therapeutic drugs for infectious diseases, cancer, and many ACE2-related diseases. The present review summarized the state-of-the-art radiotracers for ACE2 imaging, including their chemical design, pharmacological properties, radiochemistry, as well as preclinical and human molecular imaging findings. We also discussed the advantages and limitations of the currently developed ACE2-specific radiotracers.

## 1. Introduction

The renin-angiotensin system (RAS) plays a crucial role in the physiology and pathophysiology of cardiovascular diseases [1]. Furthermore, RAS is essential in regulating the function of multiple organs, including the lung, kidney, liver, and heart, as well as involvement in inflammatory responses [2]. Angiotensin-converting enzyme (ACE) and its newly identified homolog, angiotensin-converting enzyme-2 (ACE2), are two key signaling enzymes in the RAS, while ACE2 mediates vasorelaxation and counterbalances the role of ACE, which promotes vasoconstriction.

ACE2 receives increasing research attention due to its critical role in the SARS pandemic. In 2003, ACE2 was identified as a functional receptor for the SARS coronavirus [3]. Furthermore, ACE2 also has been found to be the SARS-CoV-2 entry receptor through computational modeling and viral infection experiments [4,5,6,7,8,9]. The SARS-CoV-2 virus enters human cells through the ACE2 receptor and replicates within the cells. Moreover, cryogenic electron microscopy experiments showed that SARS-CoV-2 had 10 times greater affinity for ACE2 than SARS-CoV, which may explain the global outbreak and much higher liability of SARS-CoV-2 infection [10]. The COVID-19 pandemic caused by SARS-CoV-2 has upended the daily lives of individuals around the world, with historic numbers of cases and deaths. According to early reports, COVID-19 caused acute respiratory distress syndrome and led to high morbidity and mortality in some patients [11]. It is clear that COVID-19 is not a simple viral pneumonia but presents with unusual pathophysiological effects. Our understanding of COVID-19 is constantly progressing, giving better insight into the heterogeneous nature of its acute and long-term effects. While the World Health Organization announced that COVID-19 is no longer a public health emergency of international concern on 5 May 2023, post-COVID-19 is still impacting many people’s daily lives. Many individuals endure persistent, debilitating symptoms over months after an initial infection with SARS-CoV-2 (called long COVID) [12,13,14,15,16,17,18]. Also, the numerous virus variants have contributed to variable pathological manifestations and symptom severity [19,20]. The magnitude and direction of these effects are currently unknown. Very recently, the National Institutes of Health (NIH) opened the long COVID clinical trials to investigate potential treatments for the long-term symptoms after COVID-19 infection, including the worsening of symptoms following physical or mental exertion known as post-exertional malaise, sleep disturbances, and exercise intolerance [21].

In addition to the membrane-bound ACE2 serving as a potential therapeutic target for infectious disease [22,23,24], ACE2 is also a diagnostic and prognostic biomarker in other diseases, including pancreatic cancer [25], breast cancer [26], gallbladder cancer [27], non-small cell lung cancer [28], renal cell carcinoma [29], and hepatocellular carcinoma [30]. Furthermore, ACE2 dysfunction has been implicated in cardiovascular diseases [31], neurodegenerative diseases [32], infertility [33], inflammation [34], and liver diseases [35]. Therefore, drug development targeting ACE2 may find widespread clinical applications.

Positron emission tomography (PET) and single photon emission computed tomography (SPECT) are noninvasive and highly sensitive molecular imaging techniques that allow in vivo quantification of biochemical and pharmacological processes under healthy or diseased conditions [36,37,38]. By using radiotracers, in vivo, imaging can be achieved at the molecular level to obtain quantitative information on target engagement and the degree of receptor occupancy at a given drug dose. Accordingly, many clinical achievements have been made with several promising radiotracers in the past decades [39,40,41]. Several radioligands have been developed for “visualizing” the biomarker distribution and expression of the RAS system in physiological and/or pathological conditions [42]. Recently, new molecular probes targeting ACE2 have substantially advanced our understanding of COVID-19. While reviews on molecular imaging techniques for the RAS components, such as ACE or angiotensin II type 1 receptor (AT_1_R), have emerged [43,44,45,46,47,48], to the best of our knowledge, a comprehensive review article gathering past and present advancements regarding noninvasive molecular imaging of ACE2 by using radiotracers is very limited in the literature [49]. For this review, articles were searched using the databases PubMed Central and SciFinder, with the latest search date being June 2024. The keywords “angiotensin-converting enzyme 2” or “ACE2”, “radiotracers” or “radiopharmaceutical”, and “renin-angiotensin system” or “RAS” were entered in the search engines. This article mainly reviews the recent progress in the bench-to-bedside development of radiotracers targeting ACE2 with PET or SPECT imaging modality, particularly highlighting the radiochemical aspects and imaging outcomes.

## 2. The Renin-Angiotensin System

**Figure 1:**
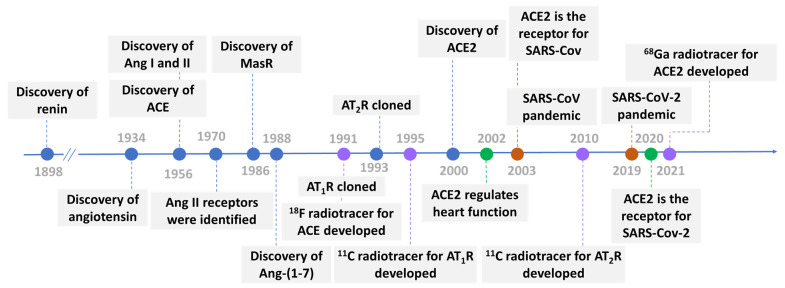
Historical timeline of discoveries and molecular imaging for the major renin-angiotensin system (RAS) system components. Discoveries of RAS components are shown in blue. Discoveries of radiotracers for RAS components are shown in purple. Discoveries of the role of ACE2 in RAS are shown in green. Coronavirus pandemics are shown in brown. ACE, angiotensin-converting enzyme; ACE2, angiotensin-converting enzymes 2; Ang, angiotensin; AT_1_R, angiotensin type 1 receptor; AT_2_R, angiotensin type 2 receptor; MasR, Mas receptor.

For over a century, since Tigerstedt et al. discovered renin in 1898 [50], the function of RAS has been known. The general knowledge of RAS has already been covered by many comprehensive reviews [51,52,53,54]. Figure 1 outlines the historical timeline for discoveries of the major components in the RAS system [55]. Studies in the 1940s revealed that constriction of the renal artery contributed to hypertension, prompting the investigation into angiotensin (hypertension and angiotonin) [56,57]. Following purification, angiotensin (Ang) was subsequently separated into two distinct forms: Ang I and Ang II [58,59]. The existence of a converting enzyme, ACE, responsible for Ang I to Ang II conversion, was predicted and subsequently isolated and characterized in 1956 [60]. The Mas receptor (MasR) and its activator Ang-(1–7) were discovered in 1986 [61] and 1988 [62,63], respectively. Angiotensin II type 2 receptor (AT_2_R), and MasR are involved in vasodilatory and anti-inflammatory responses. Both are part of the protective arm of the RAS and largely counteract the effects of the inflammation-promoted AT_1_R arm. Following this major discovery, Ang II receptors were identified as a functional entity in 1970 [64]. The instability of the solubilized receptor molecule makes for a relatively low abundance of the Ang II receptors in Ang II target tissues. AT_1_ and AT_2_ receptors were cloned in 1991 [65,66] and 1993 [67,68], respectively. In 2000, almost half a century after the discovery of ACE, ACE2 was discovered by two independent research groups [69,70]. The cardioprotective effects of ACE2 were validated in 2002 [71], shortly after the first discovery of ACE2 [69,70]. ACE2 was identified as the host receptor for SARS-CoV during the first coronavirus epidemic in 2003 [3,72]. More than 10 years later, ACE2 has garnered widespread interest as the cellular receptor of SARS-CoV-2.

The increasing understanding of RAS and the development of selective inhibitors of corresponding components also inspired the development of radiotracers for RAS. Many selective inhibitors have been discovered for RAS system components in the past. ACE inhibitors have been shown to effectively reduce high blood pressure and exert renal and cardioprotective effects. Most peptide-based inhibitors for ACE were discovered from natural resources [73,74]. The well-known small molecule ACE inhibitors, captopril, lisinopril, and enalaprilat, have proved to be clinically effective in the treatment of hypertension and congestive heart failure, but they are not inhibitors of ACE2.

A chronology of radiotracer for the RAS system is shown in Figure 1. Fluorine-18, labeled fluorocaptopril (^18^FCAP), was used to probe the ACE distribution in rats and humans in 1991 [75]. The carbon-11 labeled radiotracer MK-996, a potent nonpeptide for AT_1_R, was reported in 1995 by Mathews [76]. Since then, several carbon-11 [77,78,79,80,81,82,83,84] or fluorine-18 [85,86,87,88,89,90] AT_1_R selectively labeled PET tracers have been reported. In 2010, three carbon-11 labeled nonpeptide AT_2_R selective PET tracers using [^11^C] carbon monoxide as the labelling agent via palladium-mediated aminocarbonylation of the aryl iodide substrate were reported by Åberg [91]. The first-in-human ACE2 radiotracer was developed in 2021, over 20 years after ACE2 was discovered, to study ACE2 expression levels and distribution in healthy volunteers and a SARS-CoV-2 recovered patient (Figure 1) [92,93].

## 3. Role of ACE2 in RAS

The human ACE2 (hACE2) is a carboxypeptidase that shares 42% sequence homology with its homolog of ACE [70]. The ACE2 sequence indicates the presence of a possible signal peptide at the *N*-terminal and a hydrophobic region near the *C*-terminal, which probably functions as an anchor to the cell membrane. This results in ACE2 possessing a topology similar to ACE, which is characteristic of a type-I integral membrane protein. Unlike ACE, which has two isoforms, ACE2 has a single species of protein formed. The *C*-terminal region of ACE2 shows 48% sequence identity with collectrin, a kidney-specific transmembrane glycoprotein, and this domain does not share homology with ACE [94]. The *N*-terminal fragment of ACE2, which contains the zinc metallopeptidase catalytic site, faces the extracellular space, and this orientation makes ACE2, like ACE, an ectoenzyme capable of hydrolyzing circulating peptides. Importantly, a different in-tissue expression has been observed. ACE2 is primarily expressed in endothelial cells, arterial smooth muscle, oral mucosa, and non-vascular cells like astroglial cells and neuronal cell bodies.

**Figure 2:**
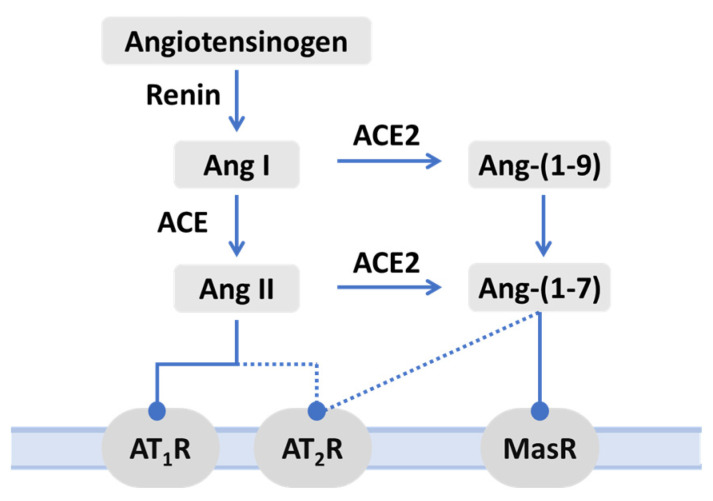
The renin-angiotensin system pathway mainly consists of angiotensin-converting enzyme ACE/angiotensin (Ang) II/angiotensin type 1 receptor (AT_1_R) axis and its counterbalancing ACE2/Ang-(1–7)/Mas axis. AT_2_R, angiotensin type 2 receptor. The dotted lines indicate the non-classical arms of the RAS.

Figure 2 shows a simplified view of ACE2 in the RAS. In the RAS, renin as a proteinase hydrolyzes angiotensinogen to Ang I, which is further cleaved by ACE to form Ang II. ACE2 not only hydrolyzes Ang I to Ang-(1–9) but also cleaves Ang II into Ang-(1–7). Compared with Ang I, Ang II is the preferred substrate of ACE2. The central role of ACE2 in RAS was consolidated a few years after its discovery by the identification of Mas as the receptor for Ang-(1–7) [95]. It is now generally accepted that the RAS comprises two arms, with ACE and ACE2 serving as the counterbalancing enzymes responsible for producing Ang II and Ang-(1–7), respectively. The classical arm encompasses ACE/Ang II/AT_1_R, whereas the alternative or protective arm consists of ACE2/Ang-(1–7)/Mas, which is implicated in numerous beneficial effects across various diseases. Also, AT_2_R is also an important part of the RAS protective axis [96,97,98]. Ang-(1–7) primarily exerts effects on the Mas receptor and, to a lesser degree, also on the AT_2_ receptor (shown in the dashed line in Figure 2).

From a functional perspective, ACE2 contributes to counterbalancing the effect of ACE [99]. Similar to ACE, ACE2 also plays significant roles in other biological processes and diseases. ACE2 provides protection against several chronic diseases, including cardiovascular diseases, lung injury, and diabetes [100,101].

## 4. Tissue Distribution of ACE2 Protein

**Figure 3:**
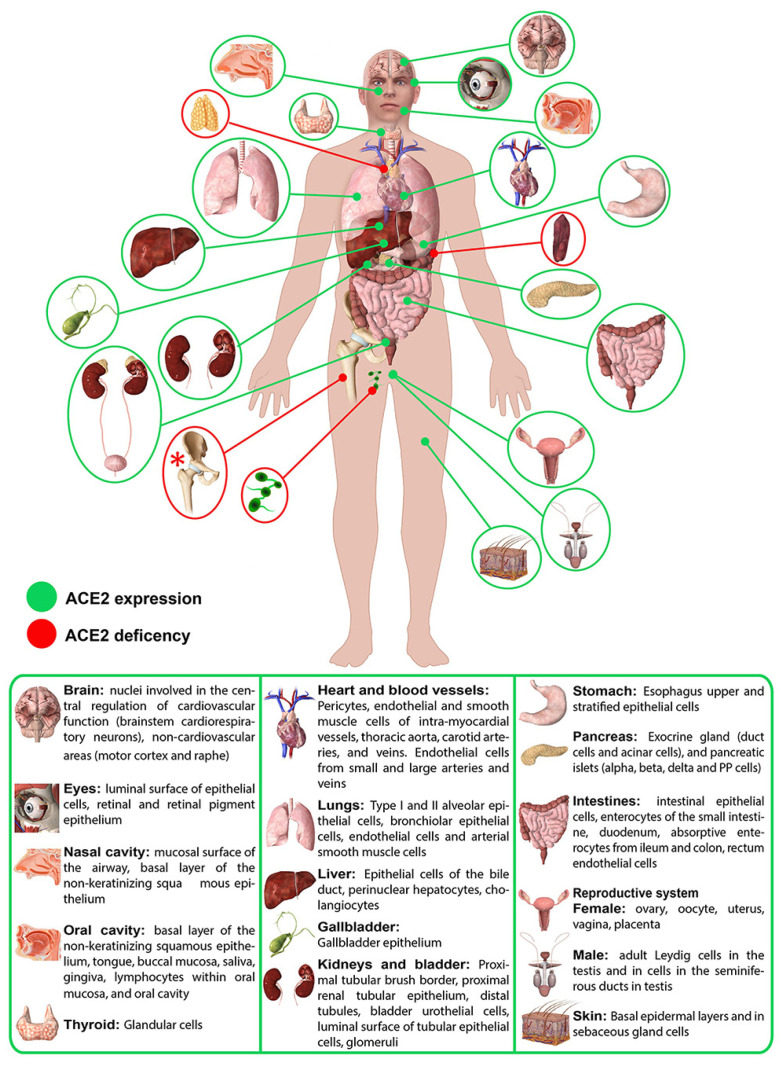
ACE2 expression in human organs. Red asterisk (*) indicates ACE2 deficiency only hypothesized. Image from Salamanna, F.; Maglio, M.; Landini, M.P.; Fini, M. Body localization of ACE-2: on the trail of the keyhole of SARS-CoV-2. Front. Med. (Lausanne). 2020, 7, 594495 is licensed under CC BY 4.0 (http://creativecommons.org/licenses/by/4.0) [107].

The expression levels of ACE2 differ between men and women due to regulation by a gene located on the X chromosome (Xp22.2), supporting the hypothesis of greater ACE2 expression in women [70]. Also, ACE2 tissue levels are regulated by oestrogens that can increase the presence of ACE2 receptors [102]. In addition, sex-based differences in attitudes and behavior may also explain the disparities in COVID-19 severity and fatality [103]. Furthermore, ACE2 mRNA is known to be present in virtually all organs, and its protein expression has become clearer over the years [104,105,106]. Figure 3 shows the ACE2 expression in human organs [107]. Recently, the highest expression of ACE2 in liver cholangiocytes, followed by hepatocytes, has been reported [108].

## 5. ACE2-Targeting Radiotracers

Radiotracers are radiolabeled compounds that are used for either diagnostic or therapeutic applications in vivo. In the case of diagnostic applications, PET and SPECT are the two major diagnostic imaging modalities in the fields of nuclear medicine and molecular imaging [109]. Both are used to visualize and diagnose diseases at the molecular and cellular levels, providing valuable information about the whole-body functional processes. The use of diagnostic radiopharmaceuticals allows for noninvasive imaging of internal organs, tissues, and bones, providing information that can be used to diagnose, stratify, and monitor the progression of diseases, such as neurological disorders and cancers [110,111,112,113,114]. The radiotracers are designed to selectively bind to specific targets in the body, allowing for visualization and quantification of their distribution and activity inside a living organism. The rational design and synthesis of selective targeted inhibitors is crucial in the development of radiotracers. Typically, radiotracer development with various radionuclides is initially inspired by existing inhibitors but aims to improve selectivity and in vivo properties. Unlike the well-known nonradioactive pharmaceuticals, diagnostic radiotracers contain very small doses of the active ingredients and often show minimal pharmacological effects. Sometimes, a good target-specific inhibitor is not necessarily a good radiotracer for clinical translations since many factors, including binding affinity, selectivity, target density, metabolic profile, pharmacokinetics, in vivo toxic effect, and dosimetry data, all contribute to decisions for further translations. Therefore, when developing new radiotracers, modification of the lead compound structures, and test-retest studies are usually carried out.

Due to the structural similarity between ACE and ACE2, the development of highly selective inhibitors is very challenging. Since ACE2 was first discovered in 2000, there have been limited ACE2-selective inhibitors reported so far [115]. Thus, the progress in developing radiotracers that specifically target ACE2 has been stalled. In 2002, Dales et al. reported a rational design approach to identify selective small-molecular ACE2 inhibitor MLN-4760 with a high binding affinity (K_i_ = 0.44 nM) and selectivity (>100 fold) against ACE [116]. In 2003, Huang et al. reported a novel ACE2-specific peptide inhibitor, DX600 (also known as cyc-DX600), which was identified by screening peptides from libraries displayed on M13 filamentous phage [117]. DX600 is a 26-amino acid cysteine disulfide-constrained peptide with a high binding affinity (K_i_ = 2.8 nM) toward ACE2. Moreover, DX600 does not inhibit ACE activity at concentrations over 100 μM. DX600 was derived from the C*_X_*P*_X_*R*_XX_*PW*_XX_*C motif with an acetylated *N*-terminal and amidated *C* terminal. The three-dimensional structure of the DX600 in water has been elucidated by proton nuclear magnetic resonance [118]. A central closed loop is formed by the constraints imposed by the intramolecular disulfide bridge. There is a basic amino acid, arginine, lying between the two prolines in the DX600. These features lead the peptide to bind ACE2 with high affinity and specificity. In addition, DX600 is chemically stable and does not hydrolyze with ACE2. Hence, it is suitable to develop micro-dose nuclear medicine molecular imaging probes by using DX600 or its analogs. In 2023, Harman et al. reported a series of constrained bicyclic peptide ACE2 inhibitors, and one of the peptides, BCY20862 (K_i_ = 0.22 nM), showed >81,000-fold selectivity for ACE2 inhibition over ACE [119]. Cyclic peptides may exhibit improved biological properties compared with linear analogs, including enhanced receptor selectivity, binding affinity, and proteolytic stability [120], because of their conformational rigidity and improved plasma stability, thus allowing for further clinical application [121,122,123]. Based on these pioneering discoveries and lead structures, we summarize recent progress for ACE2-targeted radiotracers. Within each section, we further subclassify different ACE2-specific radiotracers according to the developed imaging modalities.

### 5.1. PET Tracers Targeting ACE2

PET has emerged as a noninvasive and translational imaging technique to visualize and quantify in vivo biochemical processes in real-time. Compared with other medical imaging techniques, PET features unlimited tissue penetration, high sensitivity, better quantification, and facile animal-to-human translatability [124,125,126,127].

**Figure 4:**
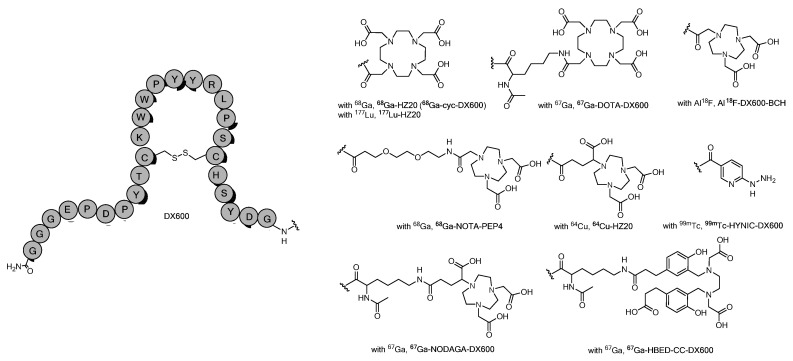
DX-600-based radiopharmaceuticals. DOTA: 1,4,7,10-tetraazacyclododecane-1,4,7,10-tetraacetic acid. NOTA: 1,4,7-triazacyclononane-1,4,7-triacetic acid. HYNIC: hydrazinonicotinic acid. NODAGA: 1,4,7-triazacyclononane,1-glutaric acid-4,7-acetic acid. HBED-CC: *N*,*N*′-bis-[2-hydroxy-5-(carboxyethyl)benzyl]ethylenediamine-*N*,*N*′-diacetic acid.

The design of ACE2-targeted PET radiotracers has attracted tremendous efforts due to the COVID-19 pandemic. To date, most of the reported radiotracers were derivatized from DX600 (Figure 4). These radiotracers were synthesized by conjugating different chelators to the *N*-terminus of DX600. The studies focus on using different linkers and a variety of PET radionuclides (^18^F, ^64^Cu, ^67^Ga, ^68^Ga).

In 2021, Wilson et al. reported the first ACE2-specific PET radiotracers (^68^Ga-NOTA-PEP) derived from DX600 [92]. Six NOTA-conjugated peptides derived from the DX600 sequence were synthesized and screened for ACE2 inhibition. Linkers are important for radiometal-based radiotracers, as the lipophilicity and stability of the linker will affect the binding affinity and selectivity of the modified peptides, and a direct coupling between the chelator and parent peptides may result in the reduction of receptor affinity. Furthermore, the linker can be utilized to adjust the pharmaceutical properties of the tracer to affect in vivo imaging results [128]. NOTA-modified cyclic peptides with various linkers showed retained or even higher ACE2 inhibition compared with parent peptide DX600 (IC_50_ = 118.2 nM with a commercially available ACE2 inhibition assays). However, NOTA-modified linear peptides without cyclic disulfide bonds showed significantly reduced activity toward ACE2. The NOTA-conjugated cyclic peptides NOTA-PEP4 with the best inhibitory activity (IC_50_ = 67.6 nM) were labeled with ^68^Ga with more than 99% radiochemical purity. The decay correction radiochemical yield of ^68^Ga-NOTA-PEP4 was 63.2 ± 6.4% (*n* = 8) with an approximate molar activity greater than 15.6 GBq/μmol. Transgenic K18-hACE2 mice expressing human ACE2 were used to validate ^68^Ga-NOTA-PEP4. An organ-specific time-activity curve demonstrated prompt clearance from the blood pool with radiotracer accumulation in the kidneys. A biodistribution and blocking study demonstrated that ^68^Ga-NOTA-PEP4 can specifically bind in the heart, liver, lungs, and intestine—organs known to be affected in SARS-CoV-2 infection (Table 1).

**Figure 5:**
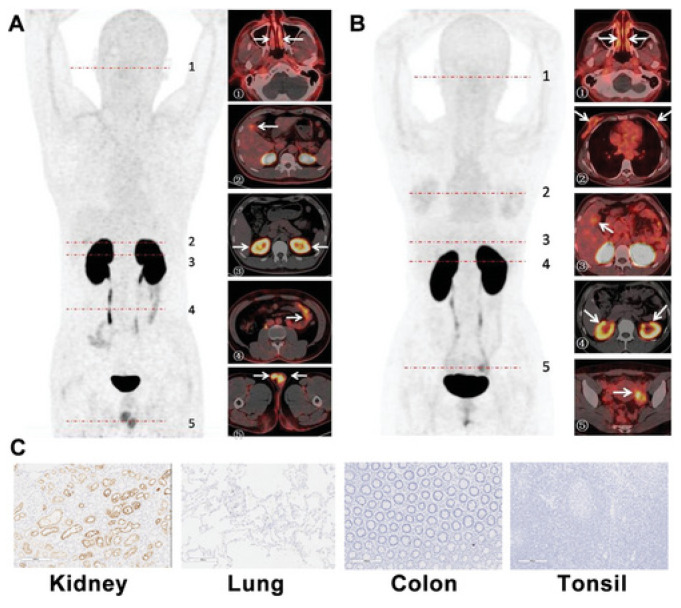
Representative positron emission tomography images of ^68^Ga-HZ20 in male and female volunteers. (**A**) Radioactivity uptake in a 38-year-old male volunteer at 90 min post-injection. 1: nasal mucosa, 2: gallbladder, 3: renal cortex, 4: small intestine, 5: testis. (**B**) Radioactivity uptake in a 34-year-old female volunteer at 90 min post-injection. 1: nasal mucosa, 2: breast, 3: gallbladder, 4: renal cortex, 5: left ovary. White arrows indicate regions of interest in (**A**,**B**). (**C**) Immunohistochemistry analysis of angiotensin-converting enzyme-2 expression in normal human organs (10×). Image from Zhu, H.; Zhang, H.; Zhou, N.; Ding, J.; Jiang, J.; Liu, T.; Liu, Z.; Wang, F.; Zhang, Q.; Zhang, Z.; et al. Molecular PET/CT profiling of ACE2 expression in vivo: implications for infection and outcome from SARS-CoV-2. Adv. Sci. 2021, 8, e2100965 is licensed under CC BY 4.0 (http://creativecommons.org/licenses/by/4.0) [93].

In the same year, Zhu et al. reported two DX600-based radiotracers labeled with ^64^Cu and ^68^Ga (^64^Cu-HZ20 and ^68^Ga-HZ20) [93]. ^64^Cu-HZ20 exhibited lower binding affinity toward ACE2 at 37 °C (143 ± 1 nM) than the parent DX600 (100 nM), which showed a higher affinity at 4 °C (66 ± 1 nM). In mouse models, both tracers showed a high accumulation in the human embryonic kidney HEK293-hACE2 xenograft mice and exhibited favorable pharmacokinetic properties in hACE2 transgenic mice. A first-in-human evaluation of ^68^Ga-HZ20 was completed in 2022 (NCT04422457). The representative PET images of ^68^Ga-HZ20 in male and female healthy volunteers are shown in Figure 5. The renal cortex, corpus luteum, and testis showed relatively high accumulation (the standardized uptake value maximum; SUV_max_ > 2.5 at 90 min post-injection). As expected for a peptide-based ligand, ^68^Ga-HZ20 cannot cross the blood-brain barrier, resulting in low accumulation of activity in the brain. Rapid elimination from the thyroid, lung, liver, spleen, adrenal gland, pancreas, and uterus suggests relatively low ACE2 expression levels, and the radioactivity gradually clears from the blood pool. However, low uptake in the lung was observed in the healthy volunteers, while the lung is one of the most critical organs for SARS-CoV-2 infection. The expression of ACE2 in the breasts of female volunteers is strongly correlated with their age, with young volunteers showing higher levels than their older counterparts.

**Figure 6:**
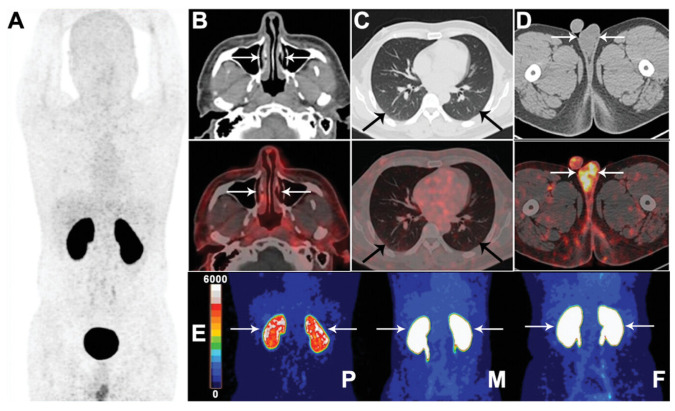
Representative positron emission tomography (PET) images of ^68^Ga-HZ20 in a volunteer recovered from COVID-19 infection. (**A**) The whole-body PET image and (**B**–**D**) axial PET/computed tomography (CT) (top panel: CT, middle panel: PET) images of nasal, lung, and testis, respectively, at 90 min post-injection were shown. (**E**) kidney uptake of ^68^Ga-HZ20 in a patient recovered from COVID (P), a healthy male (M), and a female (F) volunteer at 90 min post-injection. Arrows indicate regions of interest in (**B**–**E**). Image from Zhu, H.; Zhang, H.; Zhou, N.; Ding, J.; Jiang, J.; Liu, T.; Liu, Z.; Wang, F.; Zhang, Q.; Zhang, Z.; et al. Molecular PET/CT profiling of ACE2 expression in vivo: implications for infection and outcome from SARS-CoV-2. Adv. Sci. 2021, 8, e2100965 is licensed under CC BY 4.0 is licensed under CC BY 4.0 (http://creativecommons.org/licenses/by/4.0) [93].

It is worth mentioning that a recovered COVID-19 patient was evaluated for ^68^Ga-HZ20 uptake (Figure 6) [93]. Results show that the ^68^Ga-HZ20 accumulation in the gallbladder and testis was higher than in a control group of healthy volunteers. However, the renal cortex showed much lower uptake than healthy male and female volunteers. The findings in the recovered patient suggest that ACE2 regulation is associated with post-COVID-19 physiological changes.

Additional clinical trials are being conducted with ^68^Ga-HZ20 to gain a better understanding of the disease progression and functional recovery related to COVID-19 [133], anemia [133], and malignancies [131,132,133]. ^68^Ga-HZ20 was evaluated in healthy volunteers and patients with various disorders by Li et al. [133]. Twelve healthy volunteers without underlying diseases were recruited for whole-body PET/magnetic resonance (MR) imaging. Results showed high tracer uptake was observed in RAS-related organs (liver, spleen, and cardiac chambers). A 36-year-old man with acute COVID-19 infection underwent a PET/MR scan on the first day after symptom relief to evaluate the virus-induced changes. Compared with imaging results prior to infection, a lower ACE2 expression was observed in the liver, spleen, and testis. However, a high ACE2 expression was recorded in the upper respiratory tract, aorta, cardiac chamber, and pancreas. In post-COVID patients, diffuse higher uptake in bilateral testis was observed, which can provide molecular evidence for the current research findings that SARS-CoV-2 infection could lead to hypogonadism and testicular atrophy [141].

Compared with ^68^Ga positron-emitting radioisotopes, the ^18^F is among the most frequently used due to its unique and ideal properties [142,143,144]. Peptides can undergo direct labeling with ^18^F via Al^18^F chelation, enabling their utilization in both preclinical and clinical investigations and leveraging the accumulated knowledge and expertise from established radiometal-based PET tracers [145]. The Al^18^F-labeled Al^18^F-DX600-BCH was synthesized by Zhu et al.’s team and is currently under clinical trials (NCT04542863, expected completion in September 2024) [129,130]. It is worth noting that the uptake of Al^18^F-DX600-BCH in the lung and heart is low but is high in the nasal mucosa, possibly due to the high expression level of ACE2 in the nose.

ACE2 is also a promising target for evaluating the efficacy of COVID-19 vaccine. PET imaging can provide a more comprehensive and visual understanding of the host response post-vaccination. The high number of mutations in the viral spike protein might evade antibodies previously induced by vaccination; therefore, a broad spectrum of COVID-19 vaccines covering diverse strains is also being developed [146,147]. ^68^Ga-cyc-DX600 was utilized to study the effectiveness of the COVID-19 vaccine in a rabbit model [148]. Results showed ^68^Ga-cyc-DX600 accumulation was rebounded in the liver and spleen after a downward uptake, while the downward accumulation was not reversed in the heart, testis, and bone marrow.

It has been shown that recombinant human ACE2 (rhACE2), as a soluble supplement for human ACE2, can effectively block SARS-CoV-2 infection [149]. In 2023, Wang et al. used ^68^Ga-HZ20 to study the in vivo distribution of rhACE2 in different rhACE2 tissue and organ with in situ mouse models [135]. Imaging results showed the specific uptake of ^68^Ga-HZ20 at the rhACE2 injection site, which means this radiotracer can locate regions with different rhACE2 expression levels and reveal the distribution of rhACE2.

Spike protein is the essential envelope protein of coronavirus, which includes two subunits, S1 and S2. S1 is mainly comprised of the receptor binding domain (RBD) [150]. In the human body, the RBD binds to ACE2 on respiratory epithelial cells. The RBD could serve as an ACE2-specific binding site for radio imaging. Li et al. synthesized ^124^I-RBD to investigate ACE2 expression and distribution [137]. The PET imaging in the HepG2^ACE2+^ model nude mice showed high uptake in the lung, stomach, and thyroid. Radiolabeled nanobody ^68^Ga-Nb1159 has also been used for imaging the distribution of ACE2 in vivo [151].

Receptor recognition is an important factor determining host range and cross-species infection of viruses. The key binding site of the ACE2 receptor in pigeons has the highest coincidence (75%) with humans [152]. Therefore, pigeons were used as an animal model to explore the distribution of ACE2 in birds by using ^68^Ga-HZ20 [153]. The highest uptake in the kidney (SUV_max_ = 6.95) and lung (SUV_max_ = 1.11) was observed at 20 min post-injection. Immunohistochemical results showed high expression of ACE2 in the kidneys and small intestine, which may provide possible evidence for acute kidney injury and fecal-oral transmission of SARS-CoV-2. The lung is the primary organ infected by SARS-CoV-2. However, the probes demonstrated minimal accumulation in the lung tissue, resulting in a low SUV value, probably due to the existence of alveoli and other cavities within the lung tissue.

In addition to COVID-19-related research targeting ACE2 for aiding diagnosis, developing therapeutics, and monitoring treatment response, reduced ACE2 activity has been detected in many cancers when compared with healthy individuals [154]. The ACE2/Ang(1–7)/MasR axis in the RAS system has antitumor effects by inhibiting cancer cell proliferation and metastasis, tumor-associated angiogenesis, and epithelial-mesenchymal transition [155]. Subsequently, further research based on ^68^Ga-cyc-DX600 radiotracer was focused on different tumor models. A xenograft tumor model was used to evaluate ^68^Ga-HZ20 radiotracer [131,132]. For a patient with liver metastases from rectal cancer, ^18^F-fluorodeoxyglucose PET/CT and ^68^Ga-cyc-DX600 PET/MR imaging were conducted successively [133]. Comparatively, a significantly lower uptake of ^68^Ga-cyc-DX600 in the liver (SUV_max_ = 3.12) than its surrounding normal liver tissue (SUV_max_ = 9.34) on PET/MR imaging was observed at 75 min post-injection. The different performances between ^18^F- fluorodeoxyglucose and ^68^Ga-cyc-DX600 demonstrated the correlation of tumor activity and ACE2 expression and further validated the findings that lower ACE2 expression could be associated with higher aggressiveness of the tumor.

**Figure 7:**
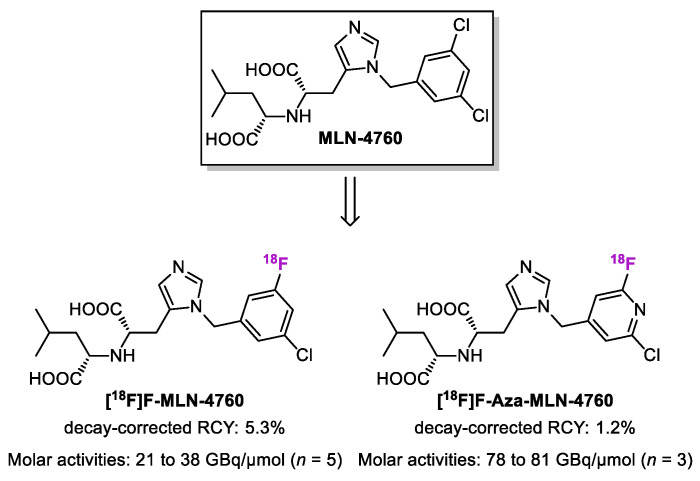
Chemical structures of MLN-4760, [^18^F]F-MLN-4760, and [^18^F]F-Aza-MLN-4760. RCY, radiochemical yield.

Very recently, Wang et al. reported two MLN-4760-based ^18^F-labeled radiotracers (Figure 7) [156]. The ACE2-binding affinities of non-radioactive reference compounds F-MLN-4760 and F-Aza-MLN-4760 were determined using HEK-ACE2 cells, with IC_50_ values of 150 nM and 387 nM, respectively. The relative ACE2-binding affinities of F-MLN-4760 and F-Aza-MLN-4760 were three-fold and seven-fold lower compared with the lead compound, MLN-4760 [116]. [^18^F]F-MLN-4760 was synthesized by Cu-mediated radiofluorination of a toxic arylstannane precursor with radiochemical purities of over 99% and a decay-corrected radiochemical yield of 5.3%. Molar activities of [^18^F]F-MLN-4760 ranged from 21 to 38 GBq/µmol (*n* = 5) at the end of the synthesis. [^18^F]F-Aza-MLN-4760 was obtained from chlorinated precursors by a halogen exchange reaction. Subsequent nucleophilic replacement of the chlorine group by [^18^F]fluoride under a high temperature (195 °C), followed by the hydrolysis of the ester moieties, afforded [^18^F]F-Aza-MLN-4760 in a radiochemical purity of over 99% with a decay-corrected radiochemical yield of up to 1.2%. Molar activities of [^18^F]F-Aza-MLN-4760 ranged from 78 to 81 GBq/µmol (*n* = 3) at the end of the synthesis, which is higher than that of [^18^F]F-MLN-4760. Both radiotracers were metabolically stable in murine and human blood plasma for up to 3 h. Similar to that previously reported by the authors, HEK-ACE2 and HEK-ACE xenograft mice were used to evaluate these tracers [138]. No radioactivity accumulation was observed in the HEK-ACE xenograft mice. Compared with the previously developed DX600-based radiopeptides, a relatively high retention of activity was seen in the gall bladder and intestinal tract in the mouse model, which could be ascribed to the rather lipophilic properties of MLN-4760-based radiotracers.

### 5.2. SPECT Tracers Targeting ACE2

SPECT imaging employs radionuclides that emit gamma rays directly, including iodine-123 and technetium-99m, which generally have lower energy and resolution than those used in PET. Generally, PET systems are more sensitive than SPECT systems. However, SPECT is more commonly used in clinics than PET because SPECT scanners are more affordable and widely available compared with PET [157].

**Figure 8:**
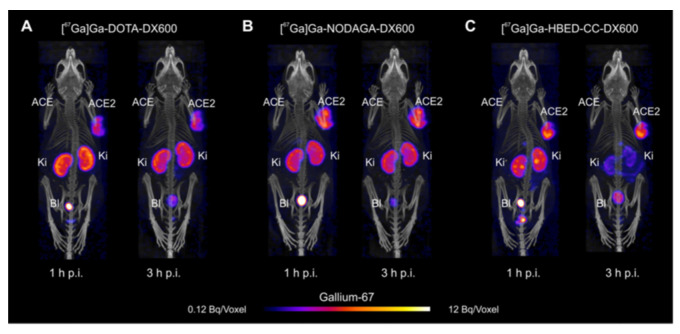
Representative single photon emission computed tomography/computed tomography images of human embryonic kidney-ACE2/ACE xenograft-bearing mice obtained 1 h and 3 h post-injection of the radiopeptides. (**A**) ^67^Ga-DOTA-DX600, (**B**) ^67^Ga-NODAGA-DX600, and (**C**) ^67^Ga-HBED-CC-DX600. Image from Beyer, D.; Vaccarin, C.; Deupi, X.; Mapanao, A.K.; Cohrs, S.; Sozzi-Guo, F.; Grundler, P.V.; van der Meulen, N.P.; Wang, J.; Tanriver, M.; et al. A tool for nuclear imaging of the SARS-CoV-2 entry receptor: molecular model and preclinical development of ACE2-selective radiopeptides. EJNMMI Res. 2023, 13, 32 is licensed under CC BY 4.0 (http://creativecommons.org/licenses/by/4.0) [138].

In 2023, Beyer reported the DOTA, NODAGA, or HBED-CC conjugated cyclic DX600 peptides for ^67^Ga (T_1/2_ = 3.26 d) chelation [138]. The selectivity of the radiolabeled peptides over ACE2 was assessed by using ACE2/ACE-transfected HEK cells and HEK-ACE2/HEK-ACE xenograft mouse model (Table 1). The labeling efficiency suggests that ^67^Ga-HBED-CC (60 MBq/nmol; radiochemical purity: 96%) has an advantage over other macrocyclic chelators, such as NODAGA and DOTA when preparing radiopeptides with high molar activities. The physicochemical properties of the three ^67^Ga labeled radiopeptides (hydrophilicity and stability) were not significantly affected by the chelators. In vitro experiments confirmed the anticipated ACE2-specific binding and uptake of these ^67^Ga-labeled radiopeptides. Biodistribution studies in HEK-ACE2 or HEK-ACE xenograft mice demonstrated similar accumulation of radioactivity in HEK-ACE2 xenografts of all three DX600-based peptides while only negligible accumulation in HEK-ACE xenografts. In normal tissues and organs, negligible accumulation of the DX600-based radiopeptide was observed in both models, which was likely attributed to the low affinity of DX600 to the murine ACE2 protein [158]. In SPECT imaging studies, results showed high radiopeptide uptake in the HEK-ACE2 xenografts at 1 h and 3 h post-injection (Figure 8). ^67^Ga-HBED-CC-DX600 demonstrated a more favorable tissue distribution profile than the other two radiopeptides with high activity accumulation in HEK-ACE2 xenografts but fast washout kinetics in the kidneys. Due to the escalating signal-to-background contrast observed over time for in vivo imaging, the acquisition at a later time point would be suitable for assessing ACE2 expression. In this regard, however, the use of the short-lived ^68^Ga (T_1/2_ = 68 min) may not be ideal; therefore, ^67^Ga (T_1/2_ = 3.26 d) would enable SPECT imaging at later time points.

The eyes are another entry site where viruses enter the body in addition to the upper respiratory tract [159]. The ocular symptoms might be the first sign of early SARS-CoV-2 infection. Li et al. reported a ^125^I-labeled pseudovirus (^125^I-CoV) used for imaging the process of viral transocular infection [139]. The labeling yield of the ^125^I-CoV was 35.1 ± 2.5%, with radiochemical purity over 95%. ACE2-specific binding affinity has been revealed to be one of the most important factors determining the infectivity of SARS-CoV-2 [160]. The transfection efficiency of the enhanced green fluorescent protein (*EGFP*) gene into ACE2-expressing HEK293T cells showed no statistical difference, indicating that I-125 labeling pseudovirus has a negligible impact on the virus’s ability to bind to the targeted ACE2 proteins. Due to the inefficient binding of SARS-CoV and SARS-CoV-2 to mouse ACE2, numerous hACE2-transgenic mouse models have been developed in past years and employed for evaluation of vaccine and therapeutic drugs as well as to elucidate the mechanisms of the SARS-CoV-2 infection process [161]. The CAG-hACE2 and ACE2-knock out (KO) mouse models were used for SPECT/computed tomography (CT) imaging by transocular inoculation of the pseudovirus, ^125^I-CoV, in the unilateral eye. The in vivo metabolism of radiotracer was dramatically different between the two animal models. A much faster metabolic clearance rate of ^125^I-CoV in ACE2-KO mice was observed. Biodistribution studies of ^125^I-CoV showed the genital glands, liver, and lungs had the highest virus concentrations. Interestingly, the genital glands had the highest unit uptake at the end of the study (72 h). This is probably due to the high expression of ACE2 in the male genital system [162]. The imaging results are helpful in understanding the transocular infection path of SARS-CoV-2, which could provide new insights into CNS symptoms ascribed to COVID-19.

In addition to the ACE2-targeting strategy using SPECT to study COVID-19-related diseases, ACE2 also serves as a promising biomarker in cancer research with SPECT imaging. Pancreatic cancer, which is associated with high morbidity and mortality rates, is one of the most aggressive malignancies. Early studies have demonstrated that ACE2 was sparsely expressed in the pancreas and was widely downregulated or absent in pancreatic cancer tissues and cells compared with the normal pancreas [25]. Very recently, Zhou et al. used DX600 peptide to conjugate it with the linker hydrazinonicotinic acid (HYNIC), followed by radiolabeling with the SPECT nuclide technetium-99m (^99m^Tc-HYNIC-DX600) [140]. The radiolabeled peptide was used to evaluate ACE2 expression with pancreatic tumor aggressiveness and growth. In the subcutaneous cell-derived xenograft models, the high tumor uptake (6.74 ± 0.31% ID/mL at 1.5 h post-injection; 3.14 ± 0.31% ID/mL at 4.5 h post-injection) was observed in the pancreas of ACE2-positive HEK293T/hACE2 mice compared with ACE2-negative HEK293T mice (1.83 ± 0.26% ID/mL at 1.5 h post-injection; 1.16 ± 0.15% ID/mL at 4.5 h post-injection).

### 5.3. Radiotherapeutics Targeting ACE2

**Figure 9:**
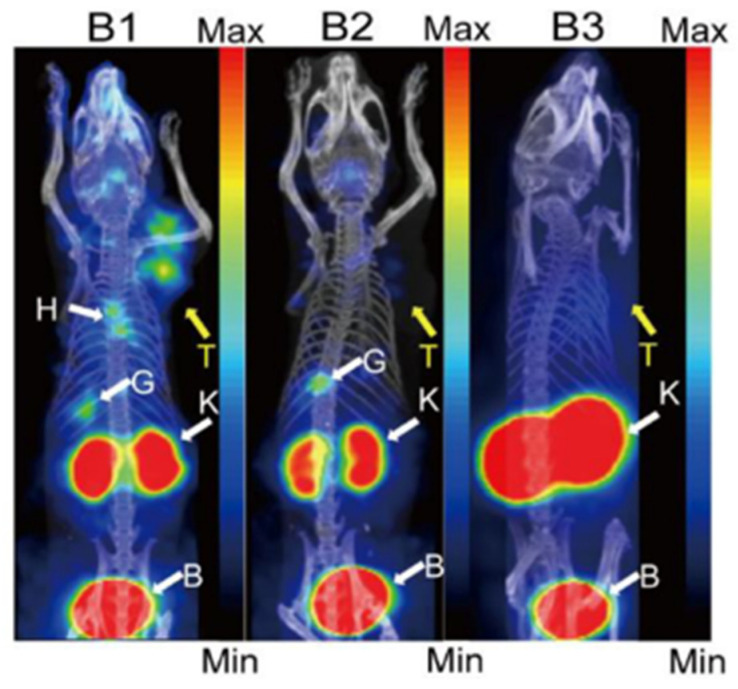
Micro-single photon emission computed tomography/computed tomography imaging of ^177^Lu-HZ20 in (B1) HepG2^ACE2+^, (B2) blocked HepG2^ACE2+^ and (B3) HepG2^WT^ tumor-bearing mice 60 min post-injection. The yellow arrow (T) indicates the tumor xenograft, the white arrow (K) indicates the kidney, (B) indicates the bladder, (H) indicates the heart, and (G) indicates the gallbladder. Image adapted from Zhang, Q.; Liu, T.; Ding, J.; Zhou, N.; Yu, Z.; Ren, Y.; Qin, X.; Du, P.; Yang, Z.; Zhu, H. Evaluation of (68)Ga- and (177)Lu-labeled HZ20 angiotensin-converting enzyme 2-targeting peptides for tumor-specific imaging. Mol. Pharm. 2022, 19, 4149–4156. Used with permission from The American Chemical Society [132].

Targeted radionuclide therapy is a novel therapeutic method for cancer. The *β^−^* particle-emitting radionuclide ^177^Lu (T_1/2_ = 159 h) is a powerful radionuclide ideal for targeted radiotherapy [163]. The success of clinical applications has been the driving force behind the development of ^177^Lu radiotracers [164,165]. ^177^Lu also possesses an imageable γ-emission, allowing quantitative SPECT imaging to determine biodistribution and radiation dosimetry. Therefore, the SPECT/CT imaging in the HepG2^ACE2+^ tumor-bearing mice model and HepG2^WT^ tumor-bearing mice were performed to evaluate ^177^Lu-HZ20 (Figure 9) [132]. The pharmacokinetics study of ^177^Lu-HZ20 showed that it had fast clearance rates through the kidney in Kunming mice (the half-lives of the biodistribution phase and elimination phase were 3.97 and 34.59 min, respectively). ^177^Lu-HZ20 showed low uptake in the brain (from 0.036 ± 0.01% ID/g at 0.5 h post-injection to 0.004 ± 0.0004% ID/g at 48 h post-injection). The radiation dosimetry of ^177^Lu-HZ20 in a male adult was estimated based on the biodistribution data in normal male mice. The dose absorbed by each organ was notably low, with the effective dose of ^177^Lu-HZ20 determined to be 6.96 × 10^−2^ mSv/MBq. 

## 6. Conclusions and Future Perspectives

The outbreak of SARS-CoV-2, with its high fatality rate in early pandemics, has sparked worldwide concern and was officially declared a global pandemic by the World Health Organization in 2020 until 2023. The rapid climb in new infections and continuing mutation of the virus prompted scientists worldwide to prioritize the discovery and advancement of novel treatments for COVID-19. Significant progress has been made in the diagnosis and treatment of COVID-19 and preventative vaccine development to adapt to the evolving variants. However, new methods of battling SARS-CoV-2 variants and long COVID are still needed in the future.

ACE2 is a cell-surface receptor that plays a critical role in the pathogenesis of SARS-CoV-2 infection. Molecular imaging for ACE2 has emerged as a valuable tool for better understanding the mechanisms of SARS-CoV-2 infection, the potential roles of ACE2 in homeostasis and diseases, and to identify potential therapeutic modulators in ACE2-related diseases. The application of both imaging and radiotherapy holds significant potential for targeting sites of diseases that overexpress ACE2.

The ongoing research still shows some limitations. First, very few ACE2-targeting probes have moved from bench to clinical investigation, which may stall drug development in ACE2-related diseases. Second, most imaging probes are based on a cyclic peptide, DX600, scaffold. A more diverse molecular lead, such as small molecules, antibodies, and aptamers, is urgently needed, particularly when researchers aim to develop imaging probes to study the impact of COVID-19 on the central nervous system (CNS). Radiolabeled DX600 and its derivatives cannot cross the blood-brain barrier and typically show negligible brain uptake regardless of COVID-19 status. Therefore, small molecule-based imaging probes, such as MLN-4760 analogs, may circumvent the limitations of studying post-COVID-19 sequela in the CNS and other ACE2-related CNS disorders. In 2023, we reported the design and synthesis of fluorinated ACE2 inhibitors based on MLN-4760 as leads to PET radioligands [166]. Concomitant with preparing this manuscript, two ^18^F-labeled MLN-4760-based PET tracers were reported [156]. These emerging examples in recent years will likely stimulate more research in developing small molecular ACE2 radiotracers and further evaluating the imaging agents in animal models. Third, due to limited scaffold targeting ACE2, few studies have developed computational modeling strategies to investigate the structure-activity relationship of the new probes toward ACE2. The lack of a valid method for drug design for the relatively new ACE2 target makes the translational pathway more tedious. Our team is working on small molecule-based imaging probes targeting ACE2, and our strategy may overcome these previously discussed limitations. We believe more emerging imaging probes will be available to the research community soon for this critical ACE2 target in the RAS for developing predictive, preventive, personalized, and participatory nuclear medicine.

## Figures and Tables

**Table 1:** Radiotracers that target the ACE2.

Radioligand	Imaging Modality	Preclinical /Clinical	Experimental Model	Binding Affinity	Radiochemical Yield	Specific/Molar Activity	Stability	Reference
Al^18^F-DX600-BCH	PET	Clinical	Healthy volunteers	NR	20.4 ± 5.2% (non-decay corrected)	3.7–18.5 GBq/μmol (*n* = 15)	Stable in saline up to 4 h	Ding 2021 [129,130]
^68^Ga-HZ20 (^68^Ga-cyc-DX600)	PET	Clinical	Recovered SARS-CoV-2 infection patients, patients with tumor, healthy volunteers, ACE2 expressing HepG2 tumor-bearing mice, HEK293 xenograft mice, HEK293T/hACE2 xenograft mice, hACE2 mice, ACE2-KO mice, pigeon, rabbit	K_d_ = 66 ± 1 nM (4 °C), 143 ± 1 nM (37 °C)	59.9 ± 3.9% (non-decay corrected, *n* = 10)	6.0 × 10^4^ GBq/mmol (*n* = 3)	Stable in 0.01 M PBS solution at 37 °C up to 120 min	Zhu 2021 [93,131,132,133,134,135]
^64^Cu-HZ20	PET	Preclinical	HEK293-hACE2 xenograft mice, hACE2 transgenic mice	K_d_ = 100.0 nM	NR	NR	NR	Zhu 2021 [93]
^68^Ga-NOTA-PEP4	PET	Preclinical	K18-hACE2 transgenic mice	IC_50_ = 67.6 nM	63.2 ± 6.4% (decay corrected, *n* = 8)	Up to 15.6 GBq/μmol	NR	Parker 2021 [92]
^68^Ga-Nb1159	PET	Preclinical	SARS-CoV-2 RBD administered mice	K_d_ = 25.53 nM (RBD)	49.48 ± 3.12% (non-decay corrected)	2.74–10.99 MBq/nmol	Stable in 0.01 M PBS solution and 5% HSA up to 8 h	Liu 2022 [136]
^124^I-RBD	PET	Preclinical	HepG2^ACE2+^ tumor-bearing mice	K_d_ = 75.7 nM	83.9 ± 4.6% (non-decay corrected, *n* = 10)	25.3–28.9 GBq/nmol	Stable in saline and 5% HSA up to 120 h	Li 2022 [137]
^67^Ga-HBED-CC-DX600	SPECT	Preclinical	HEK-ACE2/HEK-ACE xenograft mice	K_d_ = 113 ± 17 nM	NR	60 MBq/ nmol	Stable in human blood plasma up to 24 h	Beyer 2023 [138]
^67^Ga-DOTA-DX600	SPECT	Preclinical	HEK-ACE2/HEK-ACE xenograft mice	K_d_ = 98 ± 10 nM	NR	20 MBq/nmol	A release of gallium-67 was observed in mouse blood plasma (~4% after 3 h and >50% after 24 h)	Beyer 2023 [138]
^67^Ga-NODAGA-DX600	SPECT	Preclinical	HEK-ACE2/HEK-ACE xenograft mice	K_d_ = 83 ± 19 nM	NR	20 MBq/nmol	Stable in human blood plasma up to 24 h	Beyer 2023 [138]
^125^I-CoV	SPECT	Preclinical	hACE2 mice, ACE2-KO mice	NR	35.1 ± 2.5%	NR	Stable in 0.01 M PBS at 4 °C up to 72 h	Li 2022 [139]
^99m^Tc-HYNIC-DX600	SPECT	Preclinical	HEK293T/hACE2 xenograft mice	NR	NR	NR	NR	Zhou 2024 [140]
^177^Lu-HZ20	Beta radionuclide therapy	Preclinical	HepG2^ACE2+^ tumor-bearing mice	NR	84.71 ± 9.75% (*n* > 10)	(17.85 ± 1.62) × 10^6^ GBq/mmol	Stable in saline up to 100 min	Zhang 2023 [132]

ACE, angiotensin-converting enzyme; ACE2, angiotensin-converting enzymes 2; hACE2, humanized ACE2; HEK, human embryonic kidney; HSA, human serum albumin; KO, knockout; NR, not reported; PBS, phosphate-buffered saline; PET, positron emission tomography; RBD, receptor-binding domain; SPECT, single photon emission computed tomography.

## Data Availability

Available from the corresponding author upon reasonable request.
